# Diagnosing Primary Hemophagocytic Lymphohistiocytosis in an Adult: A Case Report

**DOI:** 10.7759/cureus.72884

**Published:** 2024-11-02

**Authors:** Kinaya K Smith, Linzi M Hobbs, Ravi Narra

**Affiliations:** 1 Department of Internal Medicine, Medical College of Wisconsin, Milwaukee, USA; 2 Department of Hematology and Oncology, Medical College of Wisconsin, Milwaukee, USA

**Keywords:** allogenic bone marrow transplant, bone marrow biopsy, hemophagocytic lymphohistiocytosis (hlh), neurosarcoidosis, pancytopenia

## Abstract

Primary hemophagocytic lymphohistiocytosis (HLH) is a rare, often fatal immune disorder characterized by an overactivation of the immune system. This disease is more common in children but has been known to occur in the occasional adult. The criteria for diagnosis in children do not correlate well with diagnosis in adults, and the numerous variations of presentation in adults often lead to a delay in diagnosis and treatment initiation. This case report highlights an example of primary HLH in a 41-year-old female patient who experienced a delay in diagnosis and appropriate treatment due to the similarity of her early symptoms to more common conditions. Delays in diagnosis and treatment can lead to poor outcomes, including death. Thus, it is important for providers to be able to recognize the varying signs and symptoms of primary HLH in an adult to ensure treatment is initiated as early as possible in the disease course.

## Introduction

Hemophagocytic lymphohistiocytosis (HLH) is an aggressive, rare, and often fatal syndrome that results from excessive immune activation. The primary cells involved in this disease process include histiocytes, natural killer (NK) cells, and cytotoxic lymphocytes (CTL). Histiocytes are a form of tissue-resident macrophages responsible for the recognition and destruction of dead or dying cells and invading microorganisms. NK cells make up 10-15 percent of lymphocytes and are primarily responsible for the elimination of virally infected cells as well as damaged, stressed, or infected host cells. CTL, the majority of which express CD8, are activated T-lymphocytes that also circulate, search for, and eliminate infected cells.

HLH results from the uncontrolled activation of macrophages complicated by the failure of NK cells and/or CTL to eliminate the activated cells [1]. This dysregulation results in high levels of pro-inflammatory cytokines and can cause cytokine storm, a life-threatening systemic inflammatory syndrome that results in subsequent cellular destruction in various tissues [1-3]. HLH can occur as a primary or secondary disorder. Primary HLH typically occurs in early childhood and results from genetic mutations that impair the feedback regulation between NK cells, CTL, and their target cells, causing the vicious cycle of inflammation. Secondary HLH more commonly occurs in adulthood and is typically triggered by infection, malignancy, or autoimmune disease. Given that primary HLH is an already rare, primarily pediatric disorder associated with high mortality, adult-onset presentation is extremely rare. This case report is of a 41-year-old female patient with primary HLH who presented with complaints of shortness of breath, night sweats, and pancytopenia. 

## Case presentation

The patient is a 41-year-old woman with a past medical history significant for neurosarcoidosis, ductal carcinoma in situ status post right mastectomy, hypogammaglobulinemia, and pancytopenia. She presented in July 2023 with acute, severe thrombocytopenia in the setting of a chronic five-year history of pancytopenia, malaise, myalgias, dyspnea, night sweats, difficulty walking, memory changes, and recurrent falls.

**Figure 1:**
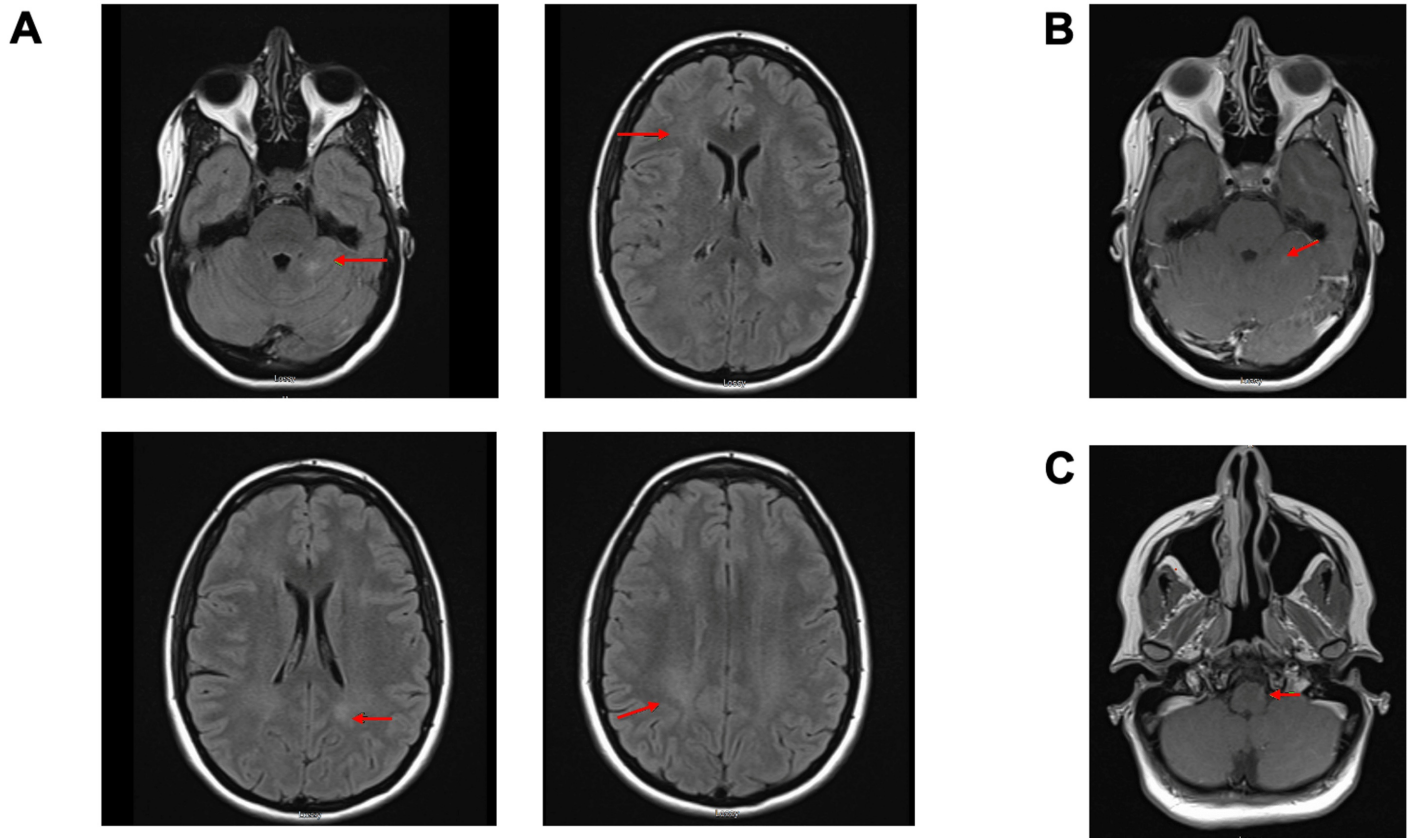
MRI brain (A) Non-enhancing fluid-attenuated inversion recovery (FLAIR) signal abnormality within the deep white matter of cerebral hemispheres marked by red arrows. (B) T1-weighted small enhancing lesion within left superior cerebellum marked by the red arrow. (C) T1-weighted linear enhancement along the left anterolateral surface of the medulla.

The patient was initially diagnosed with neurosarcoidosis in 2018 after presentation with paresthesias, vision changes, joint pain, and mobility issues. The presentation started with the development of a papular lesion on her right lower extremity that progressed to involve papular lesions on her bilateral lower extremities; she subsequently developed right lower extremity weakness and tingling of her bilateral lower extremities. She was evaluated by neurology and underwent extensive workup, including skin biopsy, MRI, CT scan, echocardiogram, and laboratory work. MRI of the brain and spine demonstrated lesions suspicious for demyelination, conus medullaris involvement, and leptomeningeal enhancement (Figure 1). A biopsy of the skin rash revealed caseating granulomas of the perineural sheath. CT chest and pulmonary function tests were normal. The echocardiogram showed normal function with no evidence of wall motion abnormalities. Laboratory studies at that time were negative for infectious etiology and autoinflammatory findings. As a result of this workup, the patient was diagnosed with neurosarcoidosis. 

The patient was initially treated with three days of plasmapheresis, with no response to treatment and progressive intolerance. She was subsequently treated with methylprednisolone followed by prednisone, with noted improvement and resolution of her bilateral lower extremity paresthesias and increased strength to her right lower extremity. Per hospital sarcoid protocol, the patient was transitioned from prednisone to infliximab in 2019; however, her disease progressed on this treatment in the form of worsening joint pain and body aches. As a result, infliximab was discontinued and she was put on prednisone for fear that her disease was steroid-dependent. The patient was started on rituximab in 2021 but had ongoing and worsening inflammation and was transitioned to cyclophosphamide in 2022. Cyclophosphamide was stopped later that year due to associated leukopenia and elevated alkaline phosphatase (ALP). 

Given some unusual features of neurosarcoidosis (i.e., absence of pulmonary parenchymal disease) and progressive disease despite appropriate treatment, additional workup was initiated to investigate further. The patient was evaluated by immunology for consideration of a possible underlying immune dysregulation and/or deficiency syndrome. The patient had no significant history of infections, and due to this and her nearly-normal IgG levels pre-steroid therapy, an immune deficiency, such as common variable immune deficiency, was deemed less likely. A monogenic disease (i.e., a disease causing immune deficiency and dysregulation as a result of a single gene mutation) was considered given her history of cytopenias, splenomegaly, and multisystemic granulomatous disease. However, this workup would have required whole exome sequencing which, under the circumstances, would not have been covered by insurance at that time, and was therefore not pursued. Reduced IgG and normal IgA/IgM in the setting of steroid therapy were noted to be consistent with steroid-induced hypogammaglobulinemia. Repeat immunoglobulin testing, off steroids, in February 2023 was notable for low IgG (199 mg/dL), elevated IgM (826 mg/dL), and normal IgA (82 mg/dL), which may have represented evolving immune deficiency (Table 1). Other workups were notable for hypercellular marrow with prominent T-cell infiltrates on initial bone marrow biopsy in 2018; eosinophilic esophagitis discovered on esophagogastroduodenoscopy (EGD) performed due to complaints for dysphagia in February 2021; and recently diagnosed right ductal carcinoma in situ discovered via mammography in January 2023 status post right mastectomy (March 2023) and tamoxifen (April 2023). 

**Table 1:** Immunoglobulin levels of the patient compared to normal immunoglobulin levels.

	Patient's values	Reference range
IgG	199 mg/dL	700-1600 mg/dL
IgM	826 mg/dL	40-230 mg/dL
IgA	82 mg/dL	70-400 mg/dL

In February 2023, the patient presented to the emergency department with complaints of acute-on-chronic chest pain, chronic dyspnea, and exertional intolerance. On admission, she was found to be pancytopenic (WBC: 2.0 10e3/uL, hemoglobin (Hgb): 8.9 g/dL, platelet (Plt): 28 10e3/uL), with prior history of chronic anemia (baseline Hgb: 8-9 g/dL) attributed to alpha thalassemia and thrombocytopenia (typically 80-100 10e3/uL) related to prior pregnancies. Hematology was consulted and a bone marrow biopsy was performed and revealed atypical T-cell infiltrates favored to be related to immune dysregulation or an autoimmune condition. Laboratory studies throughout admission were also significant for elevated transaminases (aspartate aminotransferase (AST): 87 U/L, alanine aminotransferase (ALT): 110 U/L, ALP: 1189 U/L). Hepatology was consulted and recommended a liver biopsy, which ultimately revealed mild sinusoidal lymphocytosis (predominantly T cell) without atypia, mild portal and lobular inflammation, focal extramedullary hematopoiesis, and no granulocytosis or hemophagocytosis. These findings, though peculiar, did not yield an alternative diagnosis. The original neurosarcoidosis diagnosis remained, and the patient was maintained on daily prednisone (20 mg). The plan going forward was for referral to the National Institutes of Health (NIH) to discuss possible inborn errors of immunity as the leading cause of her process; however, this referral was unable to be completed prior to her next hospitalization.

**Figure 2:**
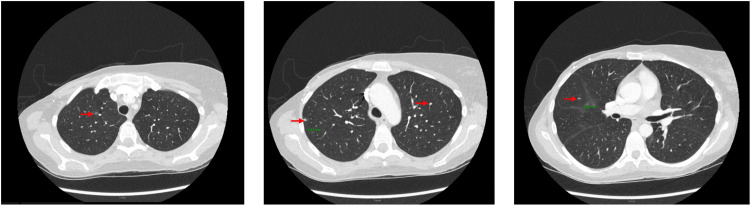
CT chest CT Chest with contrast showing pulmonary nodules throughout the lungs bilaterally, as identified by the red arrows.

**Figure 3:**
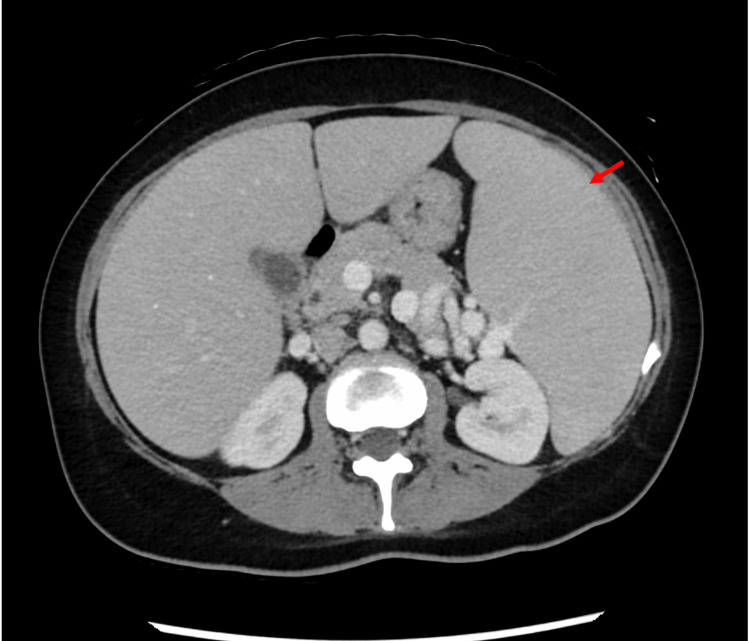
CT abdomen and pelvis CT abdomen and pelvis with contrast showing splenomegaly, as identified by the red arrow.

The patient again presented to the hospital in July 2023 due to severe thrombocytopenia (Plt: 13 10e3/uL) discovered on routine laboratory work. A review of the patient’s complicated and prolonged medical course raised concern for possible hemophagocytic lymphohistiocytosis, prompting further evaluation. During this admission, the workup was notable for persistent pancytopenia (WBC: 2.0 10e3/uL, Hgb: 9.0 g/dL, Plt: 13 10e3/uL), elevated transaminases (AST: 76 U/L, ALT: 60 U/L, ALP: 1500 U/L), and repeat bone marrow biopsy with persistent atypical T-cell infiltrates with hemophagocytic histiocytes present. Coagulation studies were abnormal (partial thromboplastin time (PTT): 35.2 sec, prothrombin time (PT): 16.3 sec, international normalized ratio (INR): 1.6, D-dimer: 0.64 mg/L, and fibrinogen: 60 mg/dL) (Table 2). Inflammatory markers were notable for elevated ferritin (1057 ng/mL), normal NK cell activity, and significantly elevated soluble IL-2 receptor (53,999 pg/mL). Epstein-Barr virus (EBV) was detected at <500 IU/mL and cytomegalovirus (CMV) was negative. CT chest, abdomen, and pelvis revealed new pulmonary nodules measuring up to 6 mm, massive splenomegaly, and no adenopathy (Figures 2, 3). A gene panel was sent and the results showed mutations in UNC13D (associated with autosomal recessive familial HLH), PMM2, and NOD2 genes, confirming the diagnosis of primary HLH [4].

**Table 2:** Lab values throughout the treatment course. The day of an allogeneic unmatched donor peripheral blood stem cell transplant with FluBu2 conditioning is February 6, 2024. FluBu2 conditioning: reduced-intensity conditioning with fludarabine/busulfan

	Feb-23	Jul-23	Aug-23	Sep-23	Oct-23	Nov-23	Dec-23	Jan-24	Feb 6-24	Mar-24	Reference range
White blood cells (x10e3/uL)	2	2	0.5	1.8	3.6	2.8	1.7	2.7	0.9	3.5	3.9-11.2
Hemoglobin (g/dL)	8.9	9	7.3	10.1	13.1	10	10.6	11.3	7.6	12.4	11.3-15.1
Platelet (x10e3/uL)	28	13	42	80	123	116	120	111	76	71	165-366
Partial thromboplastin time (sec)	32.5	1500	23.4	22.5	23.6	21.3	22.2	24.4	21.7	21.4	23-30
Prothrombin time (sec)	12.9	16.3	11.4	11.2	10.6	10.4	10.4	10.7	11.5	11.4	9.5-11.8
International normalized ratio (INR)	1.2	1.6	1.1	1.1	1	1	0.9	1	1.1	1.1	-
D-dimer (mg/L)	1.13	0.64	0.92	0.63	0.45	0.38	0.5	0.48	-	0.2	0-0.69
Fibrinogen (mg/dL)	121	60	122	93	128	131	114	159	-	93	166-438
Ferritin (ng/mL)	1582	1057	512	-	-	-	387	-	299	-	12-240
Aspartate aminotransferase(U/L)	87	76	19	21	21	19	20	16	11	22	< 35
Alanine aminotransferase (U/L)	110	60	36	50	41	31	19	14	8	32	< 30
Alkaline phosphatase (U/L)	1189	1500	64	55	63	66	69	71	58	75	35-104

Treatment of primary HLH is based on the HLH-94 protocol, which consists of eight weeks of induction therapy with etoposide, dexamethasone, and intrathecal methotrexate for those with central nervous system (CNS) involvement. The patient was initiated on this protocol after a confirmed diagnosis in July 2023 with consultation placed to our bone marrow transplant team for curative intent. Cerebrospinal fluid (CSF) obtained during lumbar puncture, performed for administration of intrathecal chemotherapy, revealed rare hemophagocytic histiocytes. The patient received a total of six rounds of intrathecal chemotherapy with eventually negative CSF studies. Given initial CSF findings, the patient remained on dexamethasone in addition to etoposide for maintenance therapy throughout December 2023. Having successfully undergone screening, workup, and donor matching, the patient was admitted in January 2024 for conditioning therapy (busulfan and fludarabine) and an allogeneic bone marrow transplant (Table 3).

**Table 3:** Diagnostic and treatment course. HLH: hemophagocytic lymphohistiocytosis

	2018	2019	2021	2022	Feb-23	Jul-23-Dec-23	Jan-24
Diagnosis	Neurosarcoidosis					Primary HLH	
Symptoms	Paresthesias, vision changes, joint pain, mobility issues				Acute-on-chronic chest pain, chronic dyspnea, and exertional intolerance		
Treatment	Plasmapheresis (no response), methylprednisolone, and prednisone taper	Infliximab	Rituximab	Cyclophosphamide		Etoposide, dexamethasone, intrathecal methotrexate	Allogenic bone marrow transplant with busulfan and fludarabine conditioning
Results	Resolution of paresthesias	Worsening joint pain and body aches	Ongoing inflammation	Leukopenia, elevated alkaline phosphatase			

## Discussion

Primary HLH is a rare condition with an incidence of 1.2 per million children and young adults per year [5]. However, primary HLH can present in adulthood, which is even rarer and thus makes it extremely difficult to determine an incidence. Given the high mortality associated with HLH (30-40% in children), it is important to quickly establish a diagnosis to swiftly begin treatment [5]. The criteria for children is based on guidelines from 2004, and a diagnosis requires five of the following eight criteria: (1) fever, (2) splenomegaly, (3) cytopenias in at least two peripheral blood lineages, (4) hypertriglyceridemia and/or hypofibrinogenemia, (5) hemophagocytosis in bone marrow, spleen, or lymph nodes, (6) low or absent NK activity, (7) hyperferritinemia, and (8) elevated levels of sIL-2r [6-8]. CXCL9 has also been added as part of this criteria [9].

Part of the challenge of diagnosing primary HLH in adults is that the criteria for children do not correlate well with the disease pattern in adults, since there are conditions in adults that can contribute to the above criteria without having a diagnosis of HLH. Additionally, given the rarity of the disease, there are a small number of case reports of primary HLH in adults with various presenting symptoms. Some presented with neurological symptoms including dysarthria, paresthesias, diplopia, impaired balance, hemiparesis, and cognitive impairment; while others presented with GI or respiratory symptoms [10-13]. However, a common symptom noted within all of these case reports is the development of fever prior to diagnosis. 

The patient in this case report presented with multiple symptoms involving the neurological, GI, and respiratory systems, as well as general symptoms including fever and night sweats. Given the multiple symptoms on initial presentation, it was difficult to distinguish if there was one unifying diagnosis, multiple diagnoses, or if any of the symptoms were related to the patient’s past medical history. Additionally, while the patient presented well into adulthood, the North American Consortium for Histiocytosis suggests that the severity of the genetic lesion could factor into when patients get diagnosed, with more severe mutations leading to an earlier presentation [14]. Genetic mutations can also act as risk factors for the development of HLH that could be triggered by infections [14]. An example of this is seen in a case report of primary HLH that was triggered by dengue fever [15]. 

As discussed, a delay in diagnosis leads to a delay in treatment, and this can result in fatality. Given that primary HLH is extremely rare in the adult population, it is not often included in the initial differential diagnoses during workup. Oftentimes, there are more plausible diseases and disorders to consider in patients presenting with vague (e.g., fever) or systematic (e.g., neurologic) symptoms. However, once appropriate workup and treatment have been completed for the suspected diagnoses, with a noted lack of improvement or progression of the disease, strong consideration should be given to a possible HLH diagnosis in patients with signs of immune activation and/or organ damage.

## Conclusions

Primary HLH is a rare, life-threatening immune disorder. While typically diagnosed during childhood, this disorder can present in adulthood. Given the propensity of this disorder to result in fatal outcomes, prompt diagnosis and treatment are often imperative to survival. HLH has a wide presentation profile and can often mimic the early stages of cancer and end-organ disease; as a result, there are often delays in diagnosis and treatment, as exemplified in this case. Suspicion for HLH should be raised when there is evidence of excessive immune activation, organ damage, and treatments for all previously considered causes are ineffective. With the various ways HLH can present, the diagnosis can be an elusive one to make. The treatment delay this elusiveness causes highlights the need for provider education. This case report aims to highlight yet another variation in the presentation of this rare and fatal genetic disorder.
